# Supplementary material for: Bipartite functional fractionation within the neural system for social cognition supports the psychological continuity of self versus other
Source: Cereb Cortex. Author manuscript; Available in PMC 2023 Feb 17. (PMC9930627; doi:10.1093/cercor/bhac135)
Supplement: Supplemental information [file EMS146262-supplement-Supplemental_information.pdf]

Supplemental information:
Bipartite functional fractionation within the neural system for social cognition supports the psychological continuity of self *vs.* other

Rocco Chiou[1,2] ✉, Christopher R. Cox[3], & Matthew A. Lambon Ralph[1] ✉
[1] MRC Cognition and Brain Sciences Unit, University of Cambridge, UK
[2] Wellcome Centre for Integrative Neuroimaging, University of Oxford, UK
[3] Department of Psychology, Louisiana State University, USA

✉ Correspondence to this work can be addressed to either of the authors by email:
(**RC:** rocco.chiou@ndcn.ox.ac.uk, **MALR:** Matt.Lambon-Ralph@mrc-cbu.cam.ac.uk).

Table of Contents

Supplemental Stimuli: Text presented in the task of personality assessment 2

Supplemental Results 1: Behavioural data and decoding at the primary motor area 4

Supplemental Results 2: Decoding at the ROIs derived from meta-analyses....... 5

Supplemental Results 3: Comparison with NeuroSynth-defined semantic activation 6

Supplemental Results 4: Comparison with NeuroSynth-defined self-related activation 7

Supplemental Results 5: Whole-brain search for univariate effects....... 8

Supplemental Results 6: Confusion matrix....... 9

Supplemental References 10

Supplemental Stimuli: Text presented in the task of personality assessment

Positive valence	Negative valence
Open to new ideas	Anxious about uncertainty
Stoic to adversity	Patronising in attitude
Quick to understand	Aloof to people
Careful when preparing	Hesitant in taking chances
Independent in daily life	Cynical and sceptical
Charitable to people	Dishonest in behaviour
Articulate when speaking	Extravagant when spending
Easy to deal with	Jealous of others
Forgiving of others' mistakes	Impatient while waiting
Optimistic about future	Impulsive when acting
Creative and talented	Gullible and naïve
Confident in self	Complacent in attitude
Fond of joking	Distressed too easily
Humble and self-effacing	Fond of gossiping
Mature and steady	Selfish and uncaring
Mild in temper	Ashamed about mistakes
Graceful in manner	Boastful when speaking
Compassionate to others	Fond of binge drinking
Willing to adventure	Feeling incompetent
Generous with money	Snobbish in attitude
Sincere to friends	Having hypocritical behaviour
Diligent at work/school	Self-conscious when speaking
Able to be trusted	Bitter about the past
Efficient in planning	Aggressive when arguing
Candid during communication	Unrealistic when planning
Adaptable to new situations	Aimless about future
Insightful when making decisions	Insecure in relationship
Proficient in learning languages	Regretful about the past
Cheerful in disposition	Worried about trivial things
Keen to help	Sensitive to criticisms
Able to multi-task	Demanding and controlling
Determined to accomplish	Unpredictable in temper
Neat in housekeeping	Low in self-esteem
Punctual at work/school	Too eager to show off
Proactive in action	Volatile in mood
Diplomatic when talking	Too harsh when commenting
Organised in planning	Forgetful in daily life
Experienced in travelling	Overly suspicious
Poetic in writing	Showy and attention-seeking
Popular with people	Overconfident about ability
Having great charisma	Too lazy to tidy up
Hospitable to guests	Pessimistic about future
Carefree in life	Stubborn in thinking
Courteous to people	Reckless and careless

Positive valence	Negative valence
Relaxed in mood	Opinionated in communication
Athletic and energetic	Too fearful of failure
Courageous in action	Overly frugal with money
Motivated to succeed	Unwilling to open up
Prudent when spending	Clumsy and awkward
Outgoing and approachable	Arrogant in attitude
Chatty and humorous	Unforgiving to people
Able to stay resilient	Condescending in manner
Pragmatic in problem solving	Childish and frivolous
Patient while waiting	Evasive and dodgy
Laid-back in manner	Withdrawn in social contexts
Collegial in teamwork	Narcissistic and egocentric
Satisfied with life	Rigid in thinking
Genuine in attitude	Unhappy about life
Caring to people	Fickle when planning
Able to keep promise	Sloppy in writing
Cordial in manner	Complaining too much
Perceptive in spotting errors	Feeble and ill
Eloquent when persuading	Timid in temper
Unassuming in manner	Passive in action
Even-tempered and calm	Too clingy to friends
Devoted to family	Restless under stress
Respected by others	All talk no action
Earnest in attitude	Dull and unsociable
Dedicated to goals	Too fussy when choosing
Flexible in thinking	Insincere in attitude
Agile in movement	Merciless in attitude
Tolerant to different views	Overly self-critical
Rational in thinking	Cunning in planning
Assertive when speaking	Unimaginative in thinking
Liberal in attitude	Unkind when speaking
Encouraging to people	Disheartened about life
Upright in character	Quarrelsome and irritable
Sympathetic to people	Estranged from family
Keen to learn new skills	Resentful about others
Well-read and cultured	Prejudiced in attitude

Supplemental Results 1: Behavioural data and decoding at the primary motor area

As illustrated in the figure below, assessing personality traits of the four target individuals results in comparable reaction times for the four conditions. Statistics ($F_{(3,69)}$ = 2.95, p = 0.04) showed that no post-hoc pair-wise comparisons revealed any reliable difference excpet for reacting to *Present Self* being significantly quicker than reacting to *Past Self* (p = 0.004). Also illustrated in the figure, the sum of positive ratings ('yes' to positive descriptions, plus 'no' to negative descriptions) was fewer for *Past Self* than any other individual ($F_{(3,69)}$ = 5.67, p = 0.002) while no reliable difference was found among the ratings of *Present Self*, *Mother*, and *Queen*. These results are consistent with the well-established literature that self-esteem generally increases from adolescence to middle adulthood (e.g., Orth & Robins, 2014). However, a possible influence of behavioural performance was that motoric preparation and button reactions might contaminate neural decoding. If such performance-related factors indeed impacted on neural representations, it would be most manifest in the motor cortex and potentially spill over to other reas. To ascertain whether behavioural performance affected decoding results, we conducted control analyses using the patterns of primary motor cortex (Brodmann area 4). Standardised anatomical mask of the bilateral motor cortex was derived from the Wake Forest PickAtlas toolbox (Maldjian, Laurienti, Kraft, & Burdette, 2003) and warped into each participant's native psace using SPM's reverse-normalisation parameters. Using the nerual patterns of motor area, in three analyses we trained support-vector machines to differentiate *Present Self vs. Past Self*, *Mother vs. Queen*, and the four-way classification of different persons. As illustrated in the figure below, results showed that decoding accuracy did *not* differ from chance in any analysis (all p > 0.24). Crucially, the classifer could reliably differentiate *Mother* from *Queen* (see Figure 1 of the main article), despite the fact that the two conditions' reaction times and ratings were equated, effectively refuting these factors as alternative explnations. Together, these suggest that the decoding results could not simply be driven by performance-related variables.

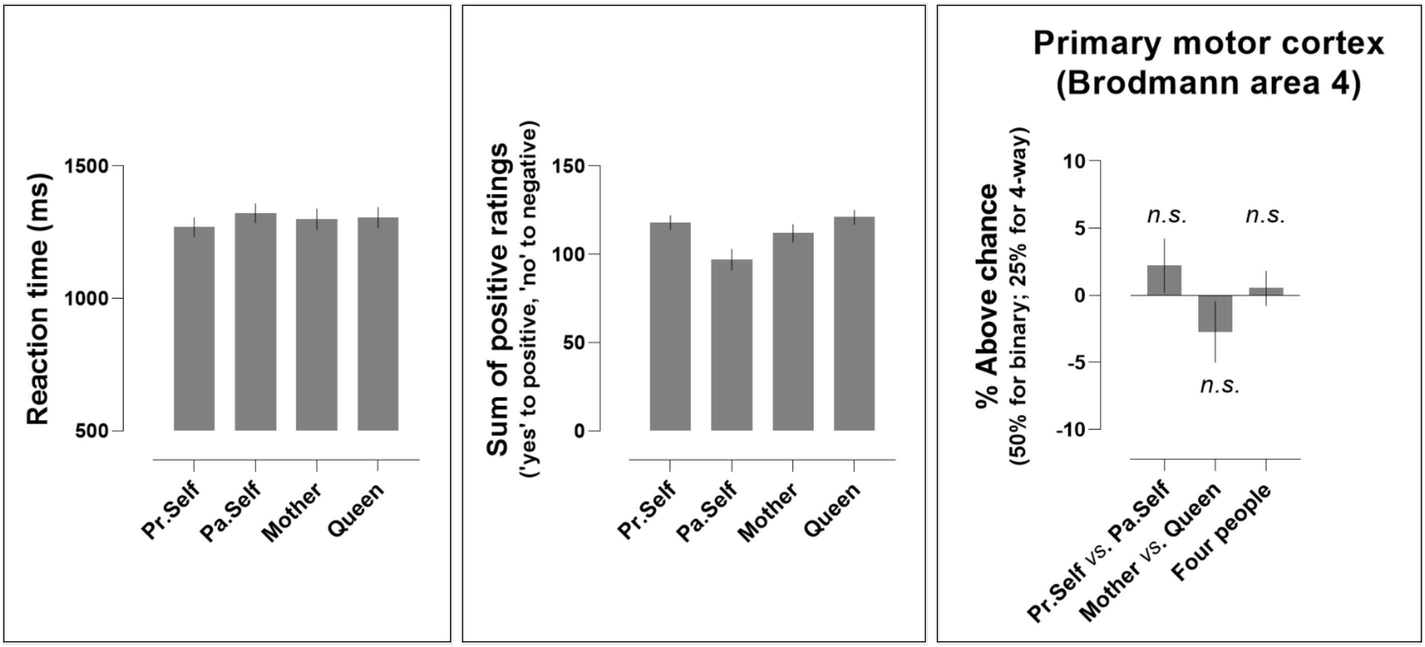

Supplemental Results 2: Decoding at the ROIs derived from meta-analyses

To ascertain that our findings do not depend the specific localiser paradigm of Dodell-Feder *et al.* (2011), we re-localised ROIs using the coordinates from three meta-analyses and performed multivoxel decoding at these literature-defined locations. For the semantic ROIs, we localised (1) the left and right anterior temporal lobes/ATLs based on Rice *et al.* (2015; see its Table 2; MNI: ±48, 8, -32) at the bilateral middle temporal gyrus and (2) the left inferior frontal gyrus/IFG based on Jackson (2020; see its Table 1) at the *pars opercularis* sub-region of the left IFG (MNI: -48, 20, 22). These two meta-analyses surveyed the fMRI literature on semantic processing and identified the bilateral ATLs and the left IFG as reliable neural regions for representing and retrieving semantic meaning, respectively. For the default-mode ROIs, we localised the dmPFC (MNI: -8, 56, 30), vmPFC (MNI: 0, 52, -12), PCC (MNI: 2, -56, 30), left IPL (MNI: -48, -56, 24), and right IPL (MNI: 56, -50, 18) using the meta-analysis of Bzdok *et al.* (2012; see its Table 1). These authors surveyed the literature of empathy and mentalising, and focused on the default-mode system. We identified corresponding points to these literature-defined coordinates in each person's native space and performed multivoxel-decoding to replicate our key results: (1) whether we could decode Present Self *vs.* Past Self, Mother *vs.* Queen, and cross-classify Self *vs.* Other across social distance and (2) whether decoding would be reliably better in the default-mode regions than semantic regions. Results below showed that the decoding based on meta-analyses are highly consistent with those based on functional localiser. Specifically, we found (1) while the ROIs were localised using a different approach, statistically robust above-chance decoding could still be detected in both semantic and default-mode ROIs and (2) decoding accuracy was consistently higher in the default-mode regions than in the semantic regions. Taken together, these additional data replicate our key results, support our conclusion, and demonstrate the robustness of our findings that they are not reliant on a specific functional localiser paradigm.

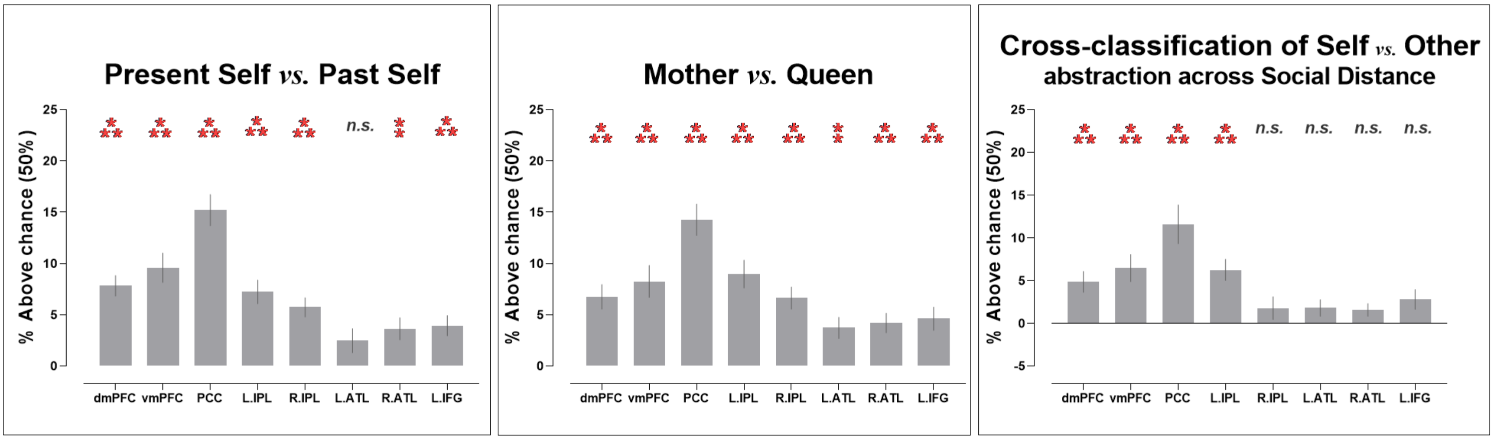

Supplemental Results 3: Comparison with NeuroSynth-defined semantic activation

The Dodell-Feder *et al.* (2011) localiser paradigm entails contrasting the Social condition against the Non-social one to delineate ROIs. Although this contrast has been able to identify clusters both in the default-mode network and in the semantic network (seen both in our data and the original study), the logic of this contrast is inherently 'social' rather than 'semantic'. To ensure that the three semantic ROIs (the left/right ATLs and the left IFG) identified with this paradigm are also reliably engaged by semantic processing, we used NeuroSynth to identify the voxels that are robustly activated by semantic tasks (based on 40,030 activations from 1,031 fMRI studies), and compared our localiser results with the NeuroSynth results. As shown in the figure below, we found that the localiser contrast was able to identify all of the three semantic ROIs, and their locations were situated within the semantically-triggered clusters of NeuroSynth (*localiser clusters*: blue, *NeuroSynth clusters*: red, *overlap*: magenta), indicating that the locations of our semantic ROIs concur with the fMRI literature of semantics.

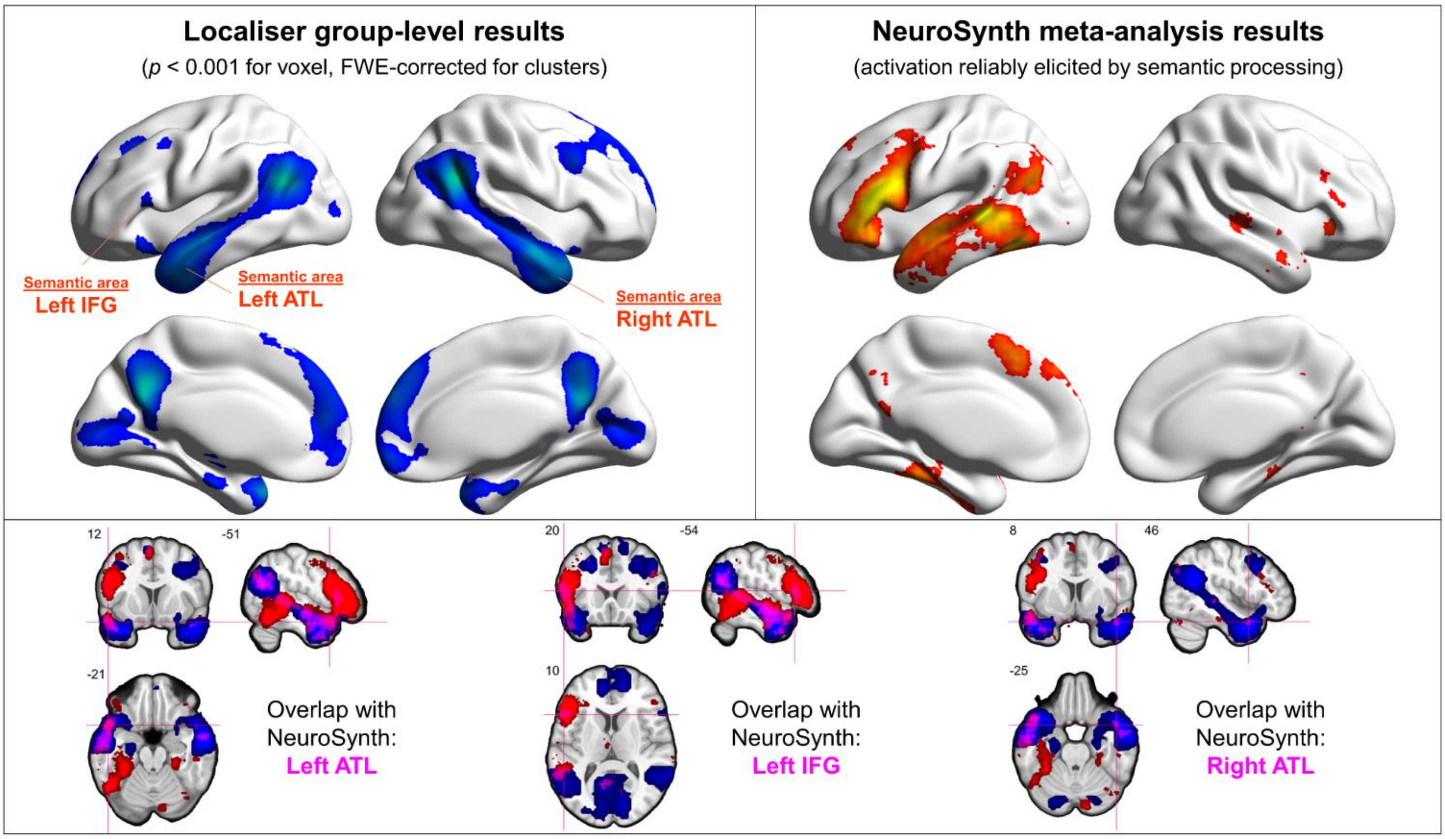

Supplemental Results 4: Comparison with NeuroSynth-defined self-related activation

While the Dodell-Feder *et al.* (2011) localiser paradigm was designed to probe 'theory of mind' (understanding another individual's mental state) and the neural basis of mentalising, it also activated various regions involved in self-referential processing. We examined the pattern of activation in the original study (Dodell-Feder *et al.*, 2011), compared it with previous studies of self-referential tasks, and found much overlap of activation (particularly in the brain's midline wall). This overlap was also observed in our own empirical results – as shown in the figure below, when comparing our localiser data with the NeuroSynth results (based on 4,728 activations from 166 studies about self-referential processing), we found substantial overlap in many regions of the default system, including the dmPFC, vmPFC, PCC, and bilateral IPL (*localiser clusters*: blue, *NeuroSynth clusters*: green, *overlap*: cyan), showing consistency between our ROIs and previous literature. This suggests that the Dodell-Feder paradigm provides an effective tool to identify broad swaths of areas in the default/semantic networks that are generally tuned to social cognition. Although there might be subtle variation between areas in terms of their tuning (e.g., the vmPFC prefers 'self' to 'other' but still reliably responds to 'other'), the localiser has been highly effective to activate the whole system that has a general proclivity to social cognition (including both 'self' and 'other').

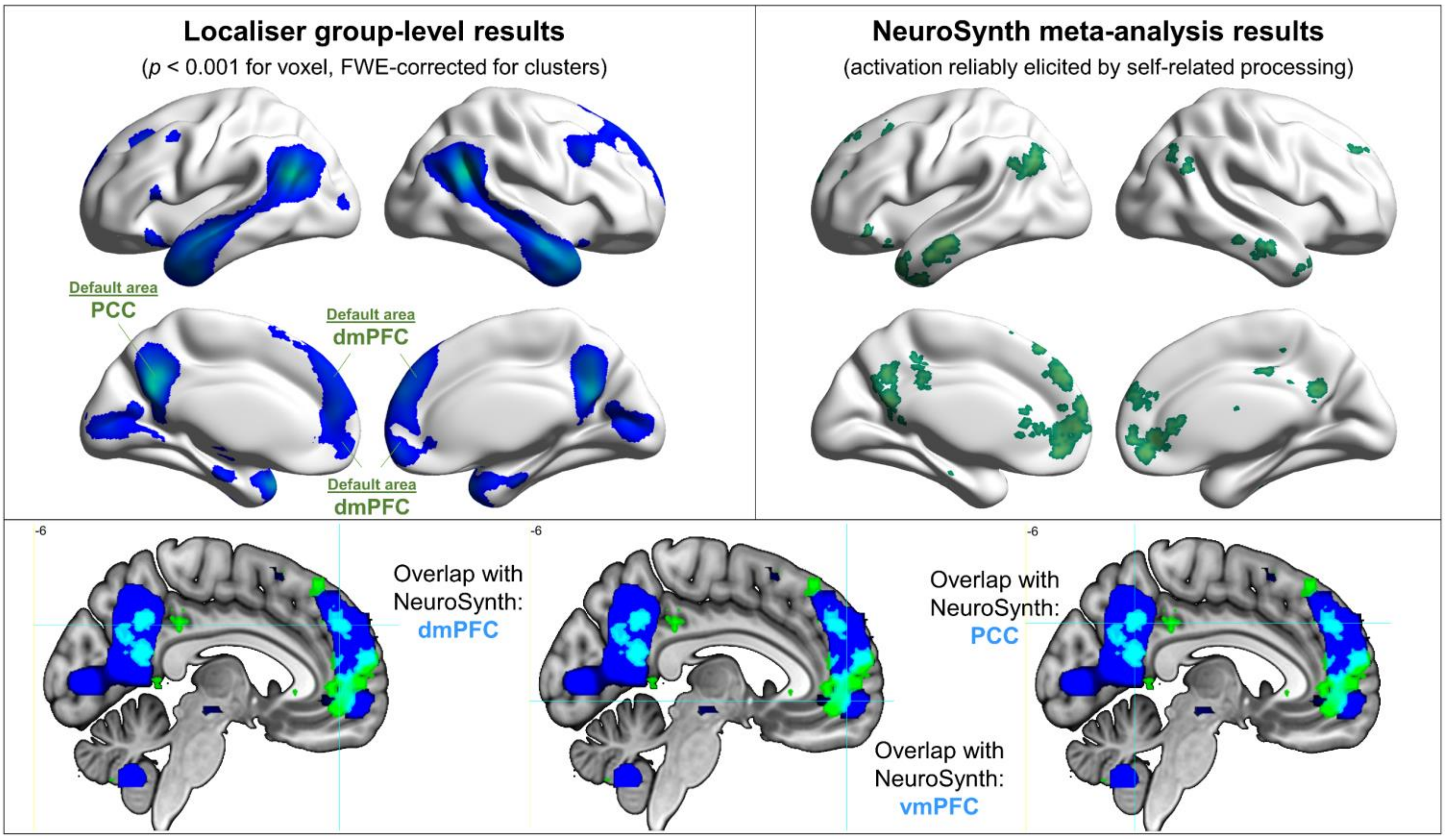

Supplemental Results 5: Whole-brain search for univariate effects

In addition to the multivariate analyses, we examined the univariate effects for three sets of contrast between conditions: Self (present & past) *vs.* Other (mother & Queen), Present Self *vs.* Past Self, Mother *vs.* Queen. Statistical threshold was $p < 0.001$ at the voxel-wise level, and FWE-correction ($p < 0.05$) for multiple comparisons at the cluster-level. Statistically reliable supra-threshold clusters were detected only in the following two comparisons: First, in the contrast of Present Self *vs.* Past Self, the rostral-medial prefrontal cortex showed greater activation for Present Self than Past Self, as indicated by the cluster in Figure (A). Second, in the comparison of Mother *vs.* Queen, three clusters were more active for Queen than Mother. As illustrated by Figure (B), these clusters are part of the default-mode network: the left/right inferior parietal lobules, and the right superior frontal gyrus.

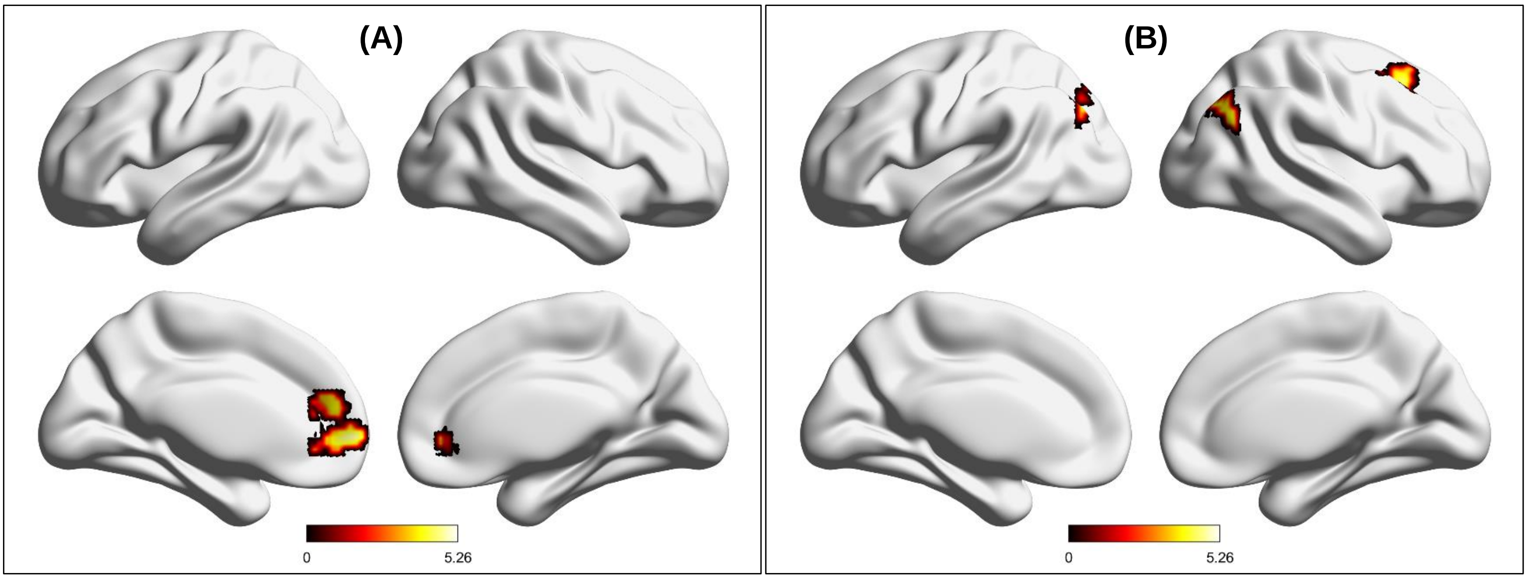

<u>Present Self > Past Self</u>

Location	Cluster size	Peak (*t*-value)	MNI Coordinate (*x*, *y*, *z*)		
Rostral-medial prefrontal cortex	258	5.26	0	47	1

<u>Queen > Mother</u>

Location	Cluster size	Peak (*t*-value)	MNI Coordinate (*x*, *y*, *z*)		
Right inferior parietal lobule	209	5.39	51	-67	31
Left inferior parietal lobule	203	5.32	-36	-79	43
Right superior frontal gyrus	165	4.88	27	23	46

<u>Past Self > Present Self</u>, **<u>Mother > Queen</u>**, **<u>Self > Other</u>**, **<u>Other > Self</u>**
No supra-threshold clusters for these contrasts

Supplemental Results 6: Confusion matrix

In this supplementary analysis, we report confusion matrices for the 4-way classification of four target individuals (*Present Self*, *Past Self*, *Mother*, and *Queen*; Figure 1A of main article), computed separately for each region of interest. The vertical axis of a matrix indicates the ground truth (actual labels), whereas the horizontal axis indicates the label predicted by the SVM classifier. The value in each cell indicates the percentage of prediction for a given pair (e.g., % of predicting 'Mother' when the actual label was indeed 'Mother'; which sums to 100% across four labels). The colour-coding depicts the classifier's performance (darker and lighter blue indicates lower and higher percentage, respectively). We observed a highly consistent pattern across all eight regions of interest. Illustrated below are the example matrices of the posterior cingulate cortex and ventromedial prefrontal cortex. It is noteworthy that the analysis shown here is a standard *cross-validation* decoding, rather than the *cross-classification* of four individuals across emotive valence (Figure 3B of main article). However, the two analyses yielded entirely coherent results – when decoding a person's identity, the algorithm was most confused with a socially proximal concept (e.g., mistaking *Present Self* as *Past Self* – more probable error) and least confused with a socially distant concept (e.g., mistaking *Present Self* as *Queen* – least probable error).

Ventromedial PFC

	PrSelf	PaSelf	Mother	Queen	sum
PrSelf	42.71	21.61	19.01	16.67	*100*
PaSelf	28.91	38.80	13.80	18.49	*100*
Mother	27.08	19.53	32.29	21.09	*100*
Queen	22.14	23.96	22.92	30.99	*100*

Posterior cingulate cortex

	PrSelf	PaSelf	Mother	Queen	sum
PrSelf	46.09	24.22	17.45	12.24	*100*
PaSelf	24.48	44.79	13.28	17.45	*100*
Mother	23.44	15.36	41.15	20.05	*100*
Queen	17.19	17.97	19.79	45.05	*100*

Supplemental References

Bzdok, D., Schilbach, L., Vogeley, K., Schneider, K., Laird, A. R., Langner, R., & Eickhoff, S. B. (2012). Parsing the neural correlates of moral cognition: ALE meta-analysis on morality, theory of mind, and empathy. *Brain Structure and Function, 217*(4), 783-796.

Dodell-Feder, D., Koster-Hale, J., Bedny, M., & Saxe, R. (2011). fMRI item analysis in a theory of mind task. *Neuroimage, 55*(2), 705-712.

Jackson, R. L. (2020). The neural correlates of semantic control revisited. *Neuroimage, 224*, 117444.

Maldjian, J. A., Laurienti, P. J., Kraft, R. A., & Burdette, J. H. (2003). An automated method for neuroanatomic and cytoarchitectonic atlas-based interrogation of fMRI data sets. *Neuroimage, 19*(3), 1233-1239.

Orth, U., & Robins, R. W. (2014). The development of self-esteem. *Current directions in psychological science, 23*(5), 381-387.

Rice, G. E., Lambon Ralph, M. A., & Hoffman, P. (2015). The roles of left versus right anterior temporal lobes in conceptual knowledge: an ALE meta-analysis of 97 functional neuroimaging studies. *Cerebral Cortex, 25*(11), 4374-4391.
